# Supplementary material for: Complex‐centric proteome profiling by SEC‐SWATH‐MS
Source: Mol Syst Biol. 2019 Jan 14;15(1):e8438. doi: 10.15252/msb.20188438 (PMC6346213; doi:10.15252/msb.20188438)
Supplement: Supplementary file 8 — Dataset EV7 [file MSB-15-e8438-s008.zip › feature_plots_string/Q9P0J6.pdf]

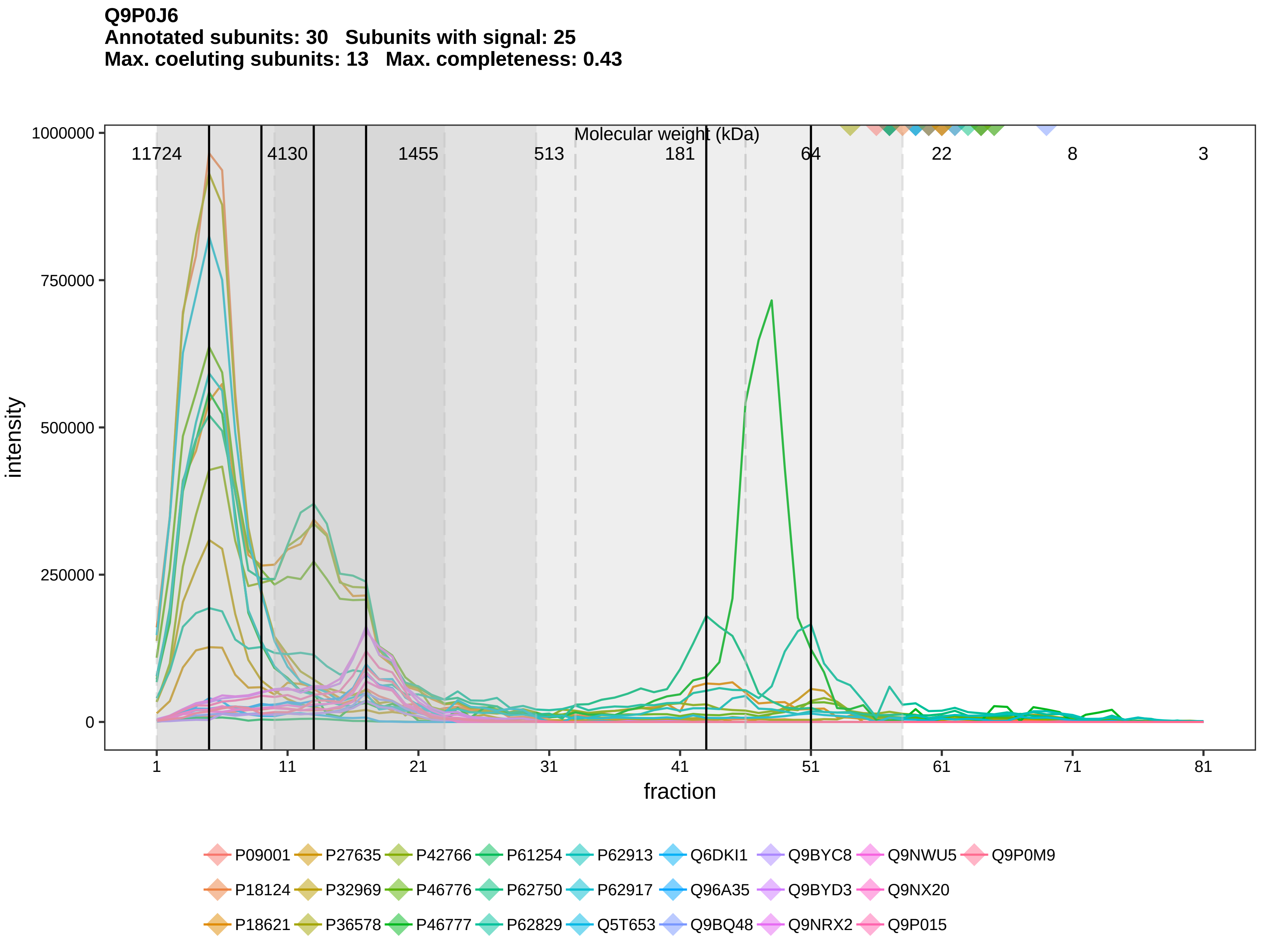
Q9P0J6
Annotated subunits: 30 Subunits with signal: 25
Max. coeluting subunits: 13 Max. completeness: 0.43
Molecular weight (kDa)
11724
4130
1455
513
181
64
22
8
3
intensity
1000000
750000
500000
250000
0
fraction
1
11
21
31
41
51
61
71
81
P09001
P27635
P42766
P61254
P62913
Q6DKI1
Q9BYC8
Q9NWU5
Q9P0M9
P18124
P32969
P46776
P62750
P62917
Q96A35
Q9BYD3
Q9NX20
P18621
P36578
P46777
P62829
Q5T653
Q9BQ48
Q9NRX2
Q9P015
